# Synthetic Flavanones Augment the Anticancer Effect of Tumor Necrosis Factor-Related Apoptosis-Inducing Ligand (TRAIL)

**DOI:** 10.3390/molecules171011693

**Published:** 2012-10-01

**Authors:** Ewelina Szliszka, Edyta Kostrzewa-Susłow, Joanna Bronikowska, Dagmara Jaworska, Tomasz Janeczko, Zenon P. Czuba, Wojciech Krol

**Affiliations:** 1 Department of Microbiology and Immunology, Medical University of Silesia, Katowice, Jordana 19, Zabrze 41-808, Poland; Email: eszliszka@sum.edu.pl (E.S.); jbronikowska@sum.edu.pl (J.B.); djaworska@sum.edu.pl (D.J.); zczuba@sum.edu.pl (Z.P.C.); 2 Department of Chemistry, Wrocław University of Environmental and Life Sciences, Norwida 25, Wrocław 50-375, Poland; Email: ekostrzew@gmail.com (E.K.-S.); janeczko13@interia.pl (T.J.)

**Keywords:** flavanones, 6-hydroxyflavanone (6-HF), 6-propionoxyflavanone (6-PF), TRAIL, apoptosis, death receptor, mitochondrial pathway, cancer cells

## Abstract

Tumor necrosis factor-related apoptosis-inducing ligand (TRAIL) is considered as the most promising anticancer agent in the TNF superfamily because of its selective cytotoxicity against tumor cells *versus* normal primary cells. However, as more tumor cells are reported to be resistant to TRAIL-mediated death, it is important to develop new therapeutic strategies to overcome this resistance. Flavonoids have been shown to sensitize cancer cells to TRAIL-induced apoptosis. The aim of this study was to examine the cytotoxic and apoptotic activities of TRAIL on HeLa cancer cells in combination with two synthetic compounds: 6-hydroxyflavanone (6-HF) and its derivative 6-propionoxy-flavanone (6-PF) and to determine the mechanism by which the flavanones overcome the TRAIL-resistance. The cytotoxicity was measured by MTT and LDH assays. The apoptosis was detected by annexin V-FITC fluorescence staining in flow cytometry and microscopy. Death receptor (TRAIL-R1/DR4 and TRAIL-R2/DR5) expression were analysed using flow cytometry. Mitochondrial membrane potential was evaluated using DePsipher staining by fluorescence microscopy. The synthetic flavanones enhanced TRAIL-induced apoptosis in HeLa cells through increased expression of TRAIL-R2 death receptor and reduction of mitochondrial membrane potential. Our study indicates that the 6-HF and 6-PF augmented the anticancer effects of TRAIL and confirm a potential use of flavanones in TRAIL-based anticancer therapy and prevention.

## 1. Introduction

**Figure 1:**
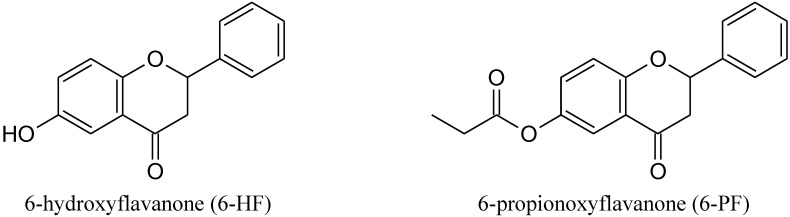
Chemical structures of tested synthetic flavanones.

Flavonoids are large class of polyphenols with a common diphenylpropanes (C6-C3-C6) structure, consisting of two aromatic rings linked through three carbons. The six major subclasses of flavonoids include flavones, isoflavones, flavonols, flavanones, flavanols and anthocyanidins [1]. These compounds have been found to possess a broad spectrum of pharmacological activities and have raised considerable interest because of their potential beneficial effects on human health. Flavonoids show antioxidant, antimicrobial, anti-inflammatory, chemopreventive and anticancer properties [1,2,3,4,5,6,7,8,9,10], therefore, the development of natural flavonoids or their synthetic derivatives is becoming increasingly recognized as an effective strategy in prevention and anticancer therapy. The induction of cancer cell-specific apoptosis via the activation of tumor necrosis factor-related apoptosis-inducing ligand (TRAIL) signaling has become an important focus of cancer research. The co-treatment of flavonoids with TRAIL might be promising as a chemoprevention and/or a new therapy against malignant tumors. In the present study we examined for the first time the cytotoxic and apoptotic effects on HeLa cancer cells of TRAIL in combination with 6-hydroxyflavanone (6-HF) or 6-propionoxyflavanone (6-PF) and explained the potential mechanism by which the two synthetic flavanones enhance TRAIL-induced apoptosis. The chemical structures of the flavanones used in the study are shown in Figure 1.

In cancer cells, activation of the apoptotic machinery by death receptor ligands of the tumor necrosis factor (TNF) superfamily of cytokines represents a novel therapeutic strategy. TRAIL, a member of TNF family, is a potent stimulator of apoptosis in a wide variety of cancer cells upon binding to pro-apoptotic receptors TRAIL-R1/DR4 and TRAIL-R2/DR5. In contrast to other members of TNF, like TNF-alpha or Fas ligand, TRAIL selectively suppresses tumor growth *in vitro* and *in vivo*, but has little or no effect on normal tissues [11,12,13,14,15]. Endogenous TRAIL expressed on the surface of immune cells or cleaved into a soluble, secreted form play an important role in the surveillance and defense against malignant tumors [16,17,18]. In recent years, numerous exogenous forms of TRAIL have been developed based on the structure and biological activities of the natural ligand. Both pre-clinical and clinical studies with recombinant human soluble TRAIL (rhsTRAIL) or TRAIL-receptor agonist (anti-TRAIL-R1 and anti-TRAIL-R2) have shown remarkable anticancer effects in a wide range of tumor types [11,12,13,14].

TRAIL triggers apoptosis in cancer cells through its interaction with specific death receptors. There are two receptors, TRAIL-R1/DR4 and TRAIL-R2/DR5, that by extracellular domains recognize and bind the ligand. The death receptors (DR) contain complete and functional intracellular death domains responsible for the activation of apoptosis pathway in cancer cells [13]. DR stimulation by TRAIL results in the recruitment of the adaptor molecule Fas-associated death domain (FADD) to form the death inducing signaling complex (DISC), which subsequently activates initiator caspase-8 and executioner caspase-3, and finally induction of apoptosis via extrinsic (receptor-dependent) pathway. Alternatively, TRAIL can also cause caspase-3 activation by affecting mitochondrial BH3-interacting domain death agonist (Bid). Crosstalk between the extrinsic and intrinsic apoptosis pathways is linked by caspase-8. Activated caspase-8 can amplify death signal via intrinsic (mitochondrial-dependent) pathway, through cleavage of Bid, along with the mitochondrial membrane potential disruption and release of cytochrome *c* [15,16,17,18]. However, some cancer cells are resistant to TRAIL-mediated apoptosis. The expression of the death receptors and proapoptotic or antiapoptotic proteins in cancer cells is involved in TRAIL-resistance [19]. TRAIL-resistant cancer cells can be sensitized to TRAIL-mediated apoptosis by certain natural and synthetic flavonoids [20,21,22,23,24,25,26,27,28,29,30,31,32,33,34,35,36,37,38,39].

## 2. Results and Discussion

### 2.1. Cytotoxic and Apoptotic Activities of Flavanones in HeLa Cancer Cells

**Figure 2:**
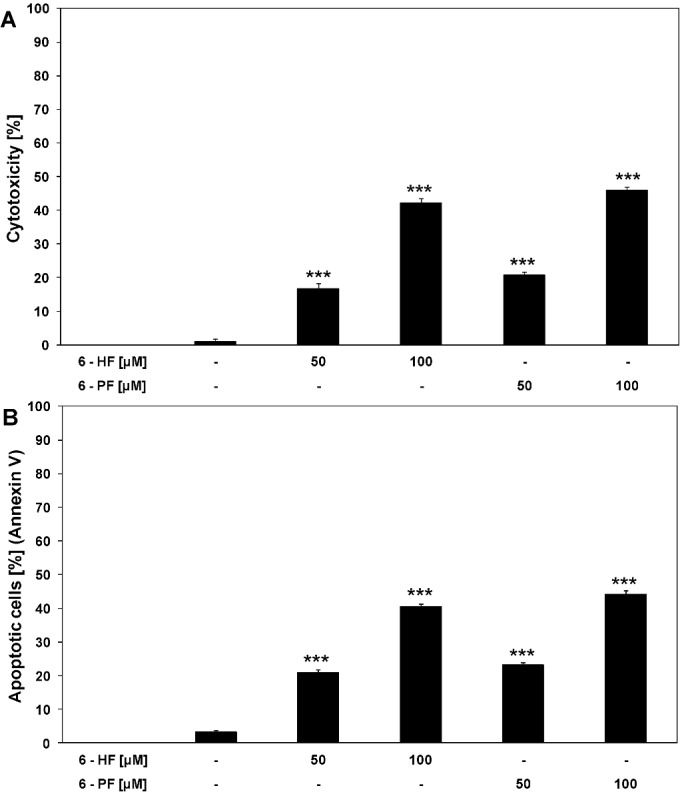
Cytotoxic and apoptotic activities of synthetic flavanones in HeLa cancer cells. The cancer cells were incubated for 48 h with 6-HF or 6-PF at the concentrations of 50 μM and 100 μM. The values represent mean ± SD of three independent experiments performed in quadruplicate (*p* < 0.05) (***p < 0.001 compared with control). (**A**) Cytotoxic activity of flavanones in HeLa cells. The percentage of cell death was measured by MTT cytotoxicity assay; (**B**) Apoptotic activity of flavanones in HeLa cells. Detection of apoptotic cell death by annexin V-FITC staining using flow cytometry.

Flavanones exhibit anti-oxidant, immunomodulatory, chemopreventive and anticancer properties [40]. Previous in vitro studies showed that naturally occurring flavanones induced cytotoxicity and apoptosis in cancer cells; naringenin in leukemia TPH1 and U937 cells [41,42], hesperidin in colon SNUC4 cells and leukemia NALM6 cells [43,44] and liquiritigenin in hepatocarcinoma SMM7721 cells and cervical carcinoma HeLa cells [45,46]. 6-HF is a synthetic compound. The flavanones with a hydroxyl groups (OH) at positions C4' and C6 have shown significant cytotoxic and apoptotic effects against tumor cells, compared with other structurally related flavanones. The hydroxylation at C6 plays an important role in anti-oxidative activity and apoptosis-inducing potential of flavanones [47]. 6-PF is a synthetic derivative of 6-HF with a propionoxyl group (C2H5COO) at the C6 position. We examined the cytotoxic and apoptotic activities of 6-HF and 6-PF against HeLa cells. Tested synthetic flavanones at concentrations of 50–100 μM induced cytotoxicity in a dose-dependent manner: 16.8 ± 1.4% to 42.1 ± 1.3% cell death for 6-HF and 20.6 ± 0.9% to 45.9 ± 0.9% cell death for 6-PF (Figure 2A). The concentrations of compounds equal 25 μM or less caused little or no anticancer effect [48]. Our results indicate that cytotoxic effects of 6-HF and 6-PF against HeLa cells were mediated through apoptosis. The percentage of necrotic cells examined by lactate dehydrogenase assay, by flow cytometry with propidium iodide and by fluorescence microscopy with Ethidium Homodimer III was near 0%. At the concentration of 50–100 μM flavanones induced apoptosis in HeLa cells in dose-dependent manner: 6-HF 20.9 ± 0.9% to 40.5 ± 0.9% and 6-PF 23.1 ± 0.7% to 44.2 ± 1.0%, respectively. The obtained results suggest that hydroxyl or propionoxyl group located at the C6 position determine the strong cytotoxic and apoptotic activities against cancer cells. In contrast to 6-PF, 6-palmitynoxyflavanone (palminynoxyl group at position C6), the other synthetic derivative of 6-HF produces no anticancer effects [48].

### 2.2. Cytotoxic and Apoptotic Activities of TRAIL in HeLa Cancer Cells

TRAIL is a death ligand and powerful inducer of apoptosis in cancer cells. Recombinant human TRAIL has been recently recommended for clinical trials in the treatment of human with neoplasm disease [11,12,13,14,15]. rhsTRAIL used in our study is a soluble protein based on a natural endogenous ligand [25]. Induction of apoptosis in cancer cells by TRAIL is a promising therapeutic approach in oncology, although TRAIL-resistance limits its efficacy [11,13,19]. We and others have demonstrated that the HeLa cell line is resistant to TRAIL-mediated death [20,25,30,49]. TRAIL at the concentration of 100 ng/mL induced 16.9 ± 1.3% cytotoxicity in HeLa cells. TRAIL caused the cytotoxic effect in cancer cells *via* the apoptotic route [30,49]. The necrotic cell death percentage of HeLa cells examined by lactate dehydrogenase assay, by flow cytometry with propidium iodide and by fluorescence microscopy with Ethidium Homodimer III was near 0%. The apoptotic activity of TRAIL at the concentration of 100 ng/mL was 19.8 ± 0.8%. TRAIL concentrations of 200 ng/mL or higher did not significantly increase the cytotoxic and apoptotic effects on HeLa cells [48].

### 2.3. Cytotoxic and Apoptotic Activities of TRAIL in Combination with Synthetic Flavanones in HeLa Cancer Cells

Several cellular mechanisms contribute to the overall anti-cancer activity of flavonoids. TRAIL-mediated apoptotic pathway is a potential target for these bioactive compounds [16]. TRAIL-resistant cancer cells can be sensitized to TRAIL-induced apoptosis by various natural and synthetic flavonoids [20,21,22,23,24,25,26,27,28,29,30,31,32,33,34,35,36,37,38,39].

**Figure 3:**
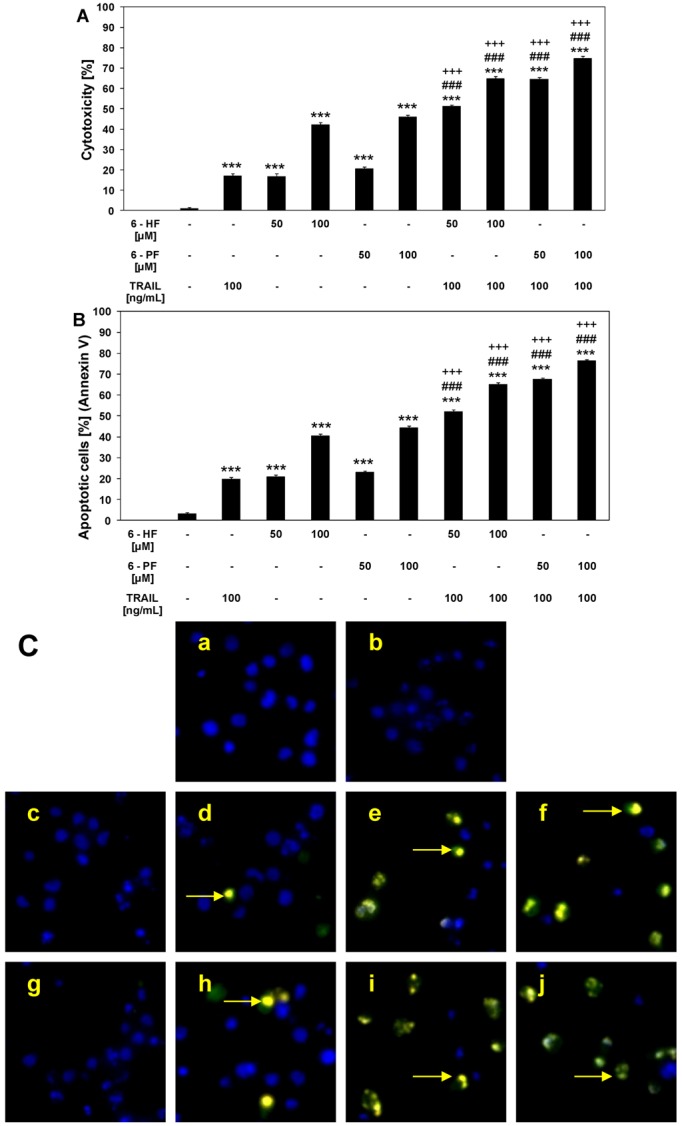
Cytotoxic and apoptotic activities of TRAIL in combination with synthetic flavanones in HeLa cancer cells. The cancer cells were incubated for 48 h with TRAIL at the concentration of 100 ng/mL and 6-HF or 6-PF at the concentrations of 50 μM and 100 μM. The values represent mean ±SD of three independent experiments performed in quadruplicate (***p < 0.001 compared with control, ^###^*p* < 0.001 compared with 6-HF or 6-PF and ^+++^*p* < 0.001 compared with TRAIL). **(A)** Co-treatment of TRAIL with flavanones induced cytotoxicity in HeLa cells. The percentage of cell death was measured by MTT cytotoxicity assay; (**B**) Co-treatment of TRAIL with flavanones induced apoptosis in HeLa cells. Detection of apoptotic cell death by annexin V-FITC and propidium iodide staining using flow cytometry; (**C**) Co-treatment of TRAIL with flavanones induced apoptosis in HeLa cells: (**a**) control cells; (**b**) cells incubated with 100 ng/mL TRAIL; (**c**) cells incubated with 50 μM 6-HF; (**d**) cells incubated with 100 μM 6-HF; (**e**) cells incubated with 100 ng/mL TRAIL and 50 μM 6-HF; (**f**) cells incubated with 100 ng/mL TRAIL and 100 μM 6-HF; (**g**) cells incubated with 50 μM 6-PF; (**h**) cells incubated with 100 μM 6-PF; (**i**) cells incubated with 100 ng/mL TRAIL and 50 μM 6-PF; (**j**) cells incubated with 100 ng/mL TRAIL and 100 μM 6-PF. Detection of apoptotic cell death by fluorescence microscopy using annexin V-FITC, Ethidium Homodimer III and Hoechst 33342 staining. The healthy cells (stained with Hoechst 33342) emitted blue fluorescence and apoptotic cells (stained with Annexin V-FITC and Hoechst 33342) emitted green and blue fluorescence (indicated by arrows). Cells undergoing apoptosis showed nuclei shrinkage, chromatin condensation and nuclei fragmentation.

We investigated the cytotoxic and apoptotic activity of TRAIL in combination with 6-HF and 6-PF on HeLa cancer cells. The cytotoxicity of TRAIL at the concentration of 100 ng/mL in combination with flavanones at the concentrations of 50 μM and 100 μM in HeLa cells was significantly increased to 51.1 ± 0.8%–64.6 ± 1.3% cell death for 6-HF and to 64.4 ± 1.1%–74.7 ± 1.1% cell death for 6-PF in comparison to TRAIL alone (Figure 3A). In contrast to 6-PF, 6-palmitynoxyflavanone, the other synthetic derivative of 6-HF, produces no anticancer effect alone or in combination with TRAIL [48]. 6-HF and 6-PF cooperate with TRAIL to induce apoptosis in cancer cells (Figure 3B). When HeLa cells were treated with the same concentrations of TRAIL and flavanones, the percentage of apoptotic cells determined by annexin V-FITC staining using flow cytometry was elevated to 52.1 ± 0.9%–65.1 ± 0.8% for 6-HF and to 67.5 ± 0.8%–76.3 ± 0.8% for 6-PF. The synthetic flavanones sensitize the TRAIL-resistant HeLa cells and markedly increase anticancer effect of death ligand. The annexin V-FITC staining visualized by fluorescence microscopy, supports the hypothesis that the apoptotic activity of TRAIL was augmented by 6-HF and 6-PF in cancer cells (Figure 3C). The exposure of cancer cells with TRAIL and flavanones for 24 h or less produced a proportionally smaller anticancer effect [48]. The necrotic cell death percentage of HeLa cells examined by lactate dehydrogenase assay, Apoptest-FITC and Apoptotic & Necrotic & Healthy Cells Quantification Kit was near zero.

Numerous findings showed that flavonoids reverse TRAIL-resistance and significantly augment the apoptotic activity of TRAIL in cancer cells [20,21,22,23,24,25,26,27,28,29,30,31,32,33,34,35,36,37,38,39]. In the flavanones subclass, naringenin enhances TRAIL-induced apoptosis in lung cancer A549 cells [36]. Many tumors remain resistant to TRAIL-mediated cell death, which is related to dominance of anti-apoptotic signals. There are many factors contributing to the resistance of cancer cells to TRAIL-induced apoptosis and flavonoids are able to overcome TRAIL-resistance [19,20,21,22,23,24,25,26,27,28,29,30,31,32,33,34,35,36,37,38,39]. Therefore, further investigations will be required to explain the molecular mechanisms by which 6-HF and 6-PF sensitize cancer cells to TRAIL-mediated death.

### 2.4. Effects of 6-HF and 6-PF on Death Receptor Expression in HeLa Cancer Cells

**Figure 4:**
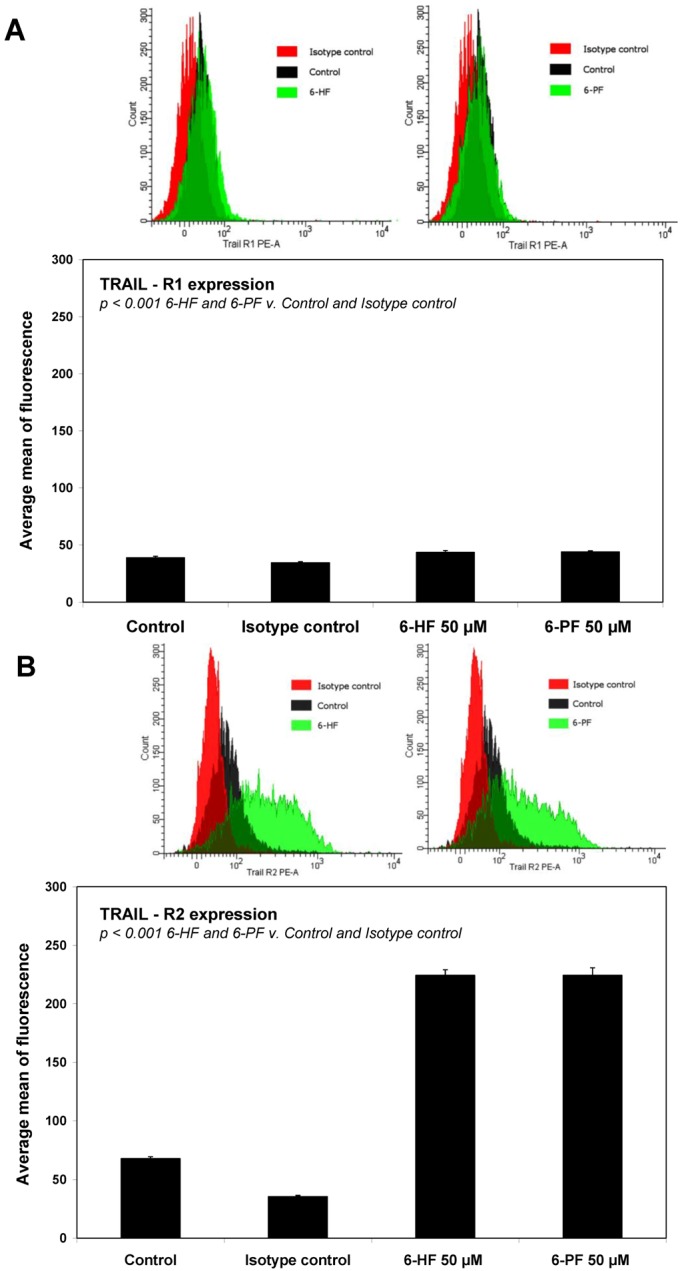
Effects of synthetic flavanones on death receptor expression in HeLa cancer cells. Cells were incubated for 48 h with 6-HF or 6-PF at the concentration of 50 μM. The surface expression of (**A**) TRAIL-R1 and (**B**) TRAIL-R2 on cancer cell was analysed by flow cytometry. The values represent mean ±SD of three independent experiments performed in quadruplicate.

TRAIL triggers cell death in various cancers through its interaction with death receptors, which contain a cytoplasmic death-domains capable of recruiting pro-apoptotic molecules and inducing apoptosis [11,15]. Expression levels of TRAIL-R1/DR4 and/or TRAIL-R2/DR5 on the cancer cell surface may play a critical role in intensity and/or duration of death receptor-mediated signaling in response to death ligands [13,19]. We determined the expression of TRAIL-R1 and TRAIL-R2 after a 48-hour incubation of HeLa cells with flavanones at the concentration of 50 μM by flow cytometry (Figure 4).

Numerous studies indicate that a lack or low expression of death receptors is involved in the resistance of cancer cells to TRAIL-mediated apoptosis [11,12,13,14,15,16,17,18,19]. Flavonoids have been demonstrated to augment TRAIL-induced apoptosis by increasing TRAIL-R2 expression in cancer cell surface. Apigenin, luteolin, baicalein, wogonin, quercetin, kaempferol, silibinin and biochanin-A influence TRAIL-R2 expression. This indicates that combinations of TRAIL and flavonoids capable of up-regulation of TRAIL-R2 may be a promising strategy for sensitization of tumors to TRAIL-induced apoptosis [20,21,22,23,24,26,27,28,37,39].

**Figure 5:**
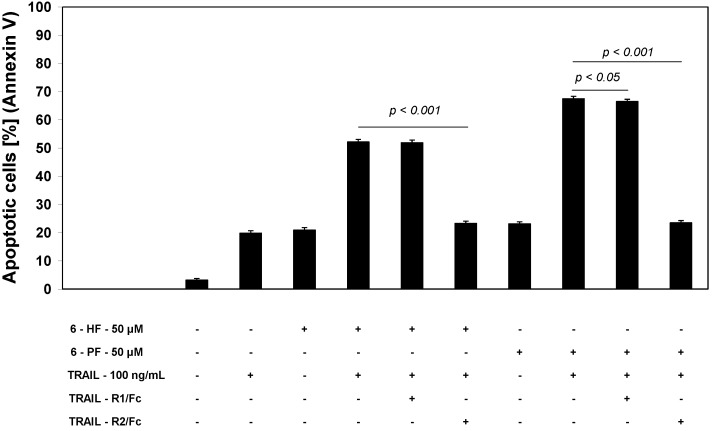
TRAIL-R2/Fc chimera block apoptosis induced by combination of TRAIL and synthetic flavanones in HeLa cancer cells. Cells were incubated for 48 h with 100 ng/mL TRAIL and/or 50 μM 6-HF or 6-PF with or without 1 μg/mL TRAIL-R1/Fc or TRAIL-R2/Fc chimera proteins. Apoptotic cell death was detected by annexin V-FITC staining using flow cytometry. The values represent mean±SD of three independent experiments performed in duplicate.

We analyzed the expression of TRAIL-R1 and TRAIL-R2 proteins in HeLa cells after treatment with synthetic flavanones. 6-HF and 6-PF significantly increased TRAIL-R2 protein levels, and a slight increase was observed in TRAIL-R1 levels post-flavanone exposure on the cell surface. To determine that the induction of apoptosis by the combination of TRAIL and 6-HF or 6-PF was mediated through TRAIL-R1 and/or TRAIL-R2, we used the TRAIL-R1/Fc or TRAIL-R2/Fc chimera protein, which has a dominant negative function against death receptors. The TRAIL-R2/Fc efficiently blocked apoptosis caused by the co-treatment of TRAIL with 6-HF or 6-PF (Figure 5). These data suggested that synthetic flavanones sensitized cancer cells to TRAIL through the extrinsic (receptor) apoptotic pathway via up-regulation of TRAIL-R2.

An increase of TRAIL-R2 expression and abrogation of TRAIL-resistance in cancer cells has been attributed to various agents, including natural or synthetic flavanones. Among flavanones, naringenin sensitized lung cancer A549 cells to TRAIL-induced apoptosis also by modulation of TRAIL-R2 protein levels [36]. Till now the mechanism underlying flavonoid-induced TRAIL-R2 up-regulation was examined by quercetin from the flavonols subclass and by luteolin from the flavones subclass. Quercetin increased TRAIL-R2 promoter activity, TRAIL-R2 mRNA and TRAIL-R2 protein levels in DU145 prostate cancer cells. TRAIL-R2 overexpression was due to both increased transcription and increased protein stability at the post-translation level by quercetin in DU145 cells. Luteolin induced TRAIL-R2 mRNA expression and increased TRAIL-R2 promoter activity in HeLa cells. This demonstrated that luteolin up-regulated TRAIL-R2 expression at the transcriptional level through TRAIL-R2 promoter in HeLa cells.

### 2.5. Effects of TRAIL and Synthetic Flavanones on Mitochondrial Membrane Potential (ΔΨm) in HeLa Cancer Cells

**Figure 6:**
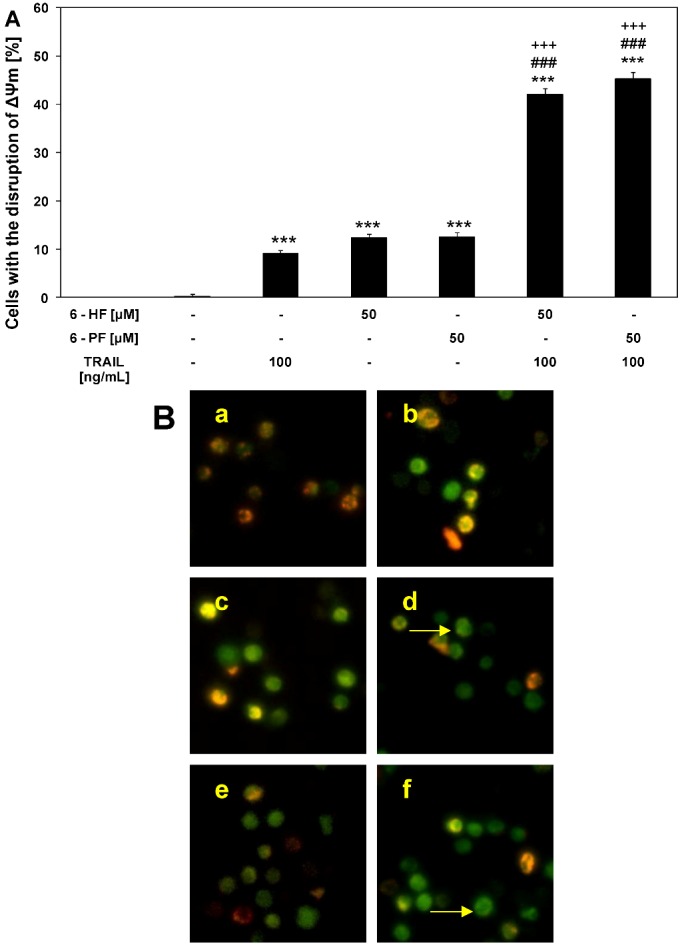
Effects of TRAIL in combination with synthetic flavanones on the mitochondrial membrane potential (ΔΨm) in HeLa cancer cells. Cells were incubated for 48 h with TRAIL at the concentration of 100 ng/mL and 6-HF or 6-PF at the concentration of 50 μM. The values represent the mean±SD of three independent experiments performed in quadruplicate (***p < 0.001 compared with control, ^###^*p* < 0.001 compared with 6-HF or 6-PF and ^+++^*p* < 0.001 compared with TRAIL). (**A**) TRAIL in combination with flavanones induced loss of ΔΨm in HeLa cells; (**B**) Disruption of ΔΨm in cancer cells was assessed by fluorescent microscopic analysis of DePsipher staining: (**a**) control cells; (**b**) cells incubated with 100 ng/mL TRAIL; (**c**) cells incubated with 50 μM 6-HF; (**d**) cells incubated with 100 ng/mL TRAIL and 50 μM 6-HF; (**e**) cells incubated with 50 μM 6-PF; (**f**) cells incubated with 100 ng/mL TRAIL and 50 μM 6-PF. Red fluorescence is emitted from the red aggregates of DePsipher which are formed within mitochondria in healthy cells. Green fluorescence reveals the monomeric form of the DePsipher molecule, which appears in the cytosol after mitochondrial membrane depolarization (indicated by arrows).

Mitochondrial membrane depolarization is one of the first intracellular changes following the onset of apoptosis [32]. The apoptosis induction by flavanones in cancer cells is associated with engagement of an intrinsic pathway. Liu *et al.* described the mitochondrial-mediated apoptosis *via* cytochrome *c* release by liquiritigenin in HeLa cells [46]. Ko *et al.* observed removal of cytochrome *c* from mitochondria to cytosol in 6-HF treated HL60 leukemic cells and demonstrated a significant role of intrinsic pathway in apoptosis caused by 6-HF [47]. We evaluated whether 6-HF and 6-PF sensitize cancer cells to TRAIL-induced mitochondrial dysfunction. The incubation of HeLa cells with 100 ng/mL TRAIL or 50 μM flavanone (6-HF and 6-PF) alone caused little effect on ΔΨm (9.1 ± 0.8%, 12.3 ± 0.9% and 12.6 ± 0.9%, respectively). The combination of TRAIL with flavanones augment the loss of ΔΨm in a large percentage of cells: 42.1 ± 1.2% for 6-HF and 45.2 ± 1.5% for 6-PF and induced a significant disruption of the ΔΨm (Figure 6). These results confirm the involvement of intrinsic (mitochondrial) apoptotic pathway in HeLa cells co-treated with TRAIL and synthetic flavanones.

Among flavonoids, quercetin also stimulated mitochondrial membrane depolarization and cytochrome *c* release in non-Hodgkin’s lymphoma B cells. This flavonol induced the depletion of Mcl-1 in VAL and RL cell lines, while other members of Bcl-2 family were not changed [50].

In many types of cancer cells the deregulation of apoptotic pathway in the course of carcinogenesis concerns both extrinsic and intrinsic pathways [11,16,18]. Jin *et al.* showed that natural flavanone naringenin enhances TRAIL-induced apoptosis in lung cancer A549 cells through increase of TRAIL-R2 expression and loss of mitochondrial membrane potential. Additionally, co-treatment of TRAIL with naringenin markedly reduced total Bid protein in A549 cells, which suggests the crosstalk between both apoptotic pathways, the extrinsic (receptor) and intrinsic (mitochondrial) [36]. We report that the augmentation of TRAIL activity by synthetic flavanones, 6-HF and 6-PF, also affects both receptor-mediated and mitochondrial signals leading to apoptosis.

## 3. Experimental

### 3.1. Chemistry

#### 3.1.1. General

Melting points (°C) were determined using a Boetius apparatus (Kofler block). The ^1^H-MNR and ^13^C-NMR spectra were recorded on a Bruker Avance DRX 300 Spectrometer. Optical rotations were measured on an automatic polarimeter JASCO P-2000-Na, equipped with an intelligent remote module (iRM) (ABL&E-JASCO, Kraków, Poland). Reactions and the resulted products were monitored by thin layer chromatography (TLC), which was carried out on silica gel 60 F254 plates (Merck, Darmstadt, Germany). Plates were visualized under UV light (were appropriate). Chromatograms were developed using the following systems: hexane: ethyl acetate (7:3), dichloromethane: ethyl acetate (1:1). Column chromatography (CC) was purchased on silica gel (230–400 mesh), a product of Merck Company. HPLC analyses were performed with a Waters 2690 instrument equipped with Waters 996 photodiode array detector, using ODS 2 column (4.6 × 250 mm, Waters) and a Guard-Pak Inserts μBondapak C18 pre-column. Separation conditions were as follows: gradient elution, using 80% of acetonitrile in 4.5% formic acid solution (eluent A) and 4.5% formic acid (eluent B); flow 1 mL/min; detection wavelength 280 nm; program: 0–7 min, 10% A 90% B; 7–10 min, 50% A 50% B; 10–13 min, 60% A 40% B; 13–15 min, 70% A 30% B; 15–20 min 80% A 20% B; 20–30 min 90% A 10% B; 30–40 min, 100% A.

6-Hydroxyflavanone (6-HF), a racemic substrate for the reaction, was purchased from Sigma Chemical Company (St. Louis, MO, USA). 6-HF: C_15_H_12_O_3_; melting point 234–235 °C; R_t_ 15.64 min (HPLC). A full description of the ^1^H-NMR and ^13^C-NMR spectra of 6-HF can be found in our previous paper [51]. 6-Propionoxyflavanone (6-PF) is a synthetic derivative of 6-HF.

#### 3.1.2. Synthesis of 6-PF

**Scheme 1:**
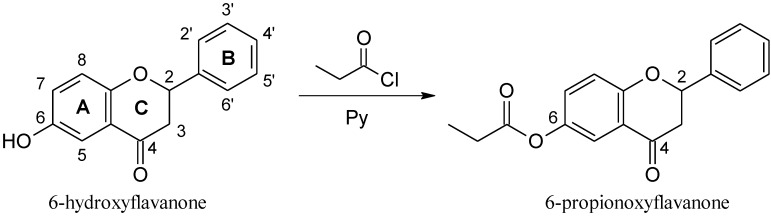
Synthesis of 6-PF.

Racemic 6-HF (25 mmol) was added to a mixture of pyridine (Py, 0.62 mmol) and propionyl chloride (0.58 mmol) in tetrahydrofuran (THF, 5 mL) at room temperature with stirring for 30 min (the progress of the reaction was monitored by TLC). Next, ethyl acetate (5 mL) was added to the reaction mixture, which was washed with HCl (0.5 M) until the solution became slightly acidic (pH = 5). Then the organic layer was separated and the aqueous layer was additionally extracted with ethyl acetate (3 × 5 mL). The combined organic layers were washed with brine until neutral and dried over anhydrous MgSO_4_. After evaporation of the solvent and purification by CC, (±)-6-PF was obtained in 94% yield (Scheme 1). The results of X-ray analysis of (±)-6-PF were presented earlier [52].

*6-Propionoxyflavanone* (6-PF, C_18_H_16_O_4_): mp. 112–115 °C; R_t_ 20.96 min (HPLC); purity 99% (HPLC); [α]: 0, (c = 1.00, CH_3_CN). ^1^H-NMR (THF-d_8_) δ: 1.19 (3H, t, *J *= 7.5 Hz, CH_3_CH_2_-), 2.58 (2H, q, *J *= 7,5 Hz, CH_3_CH_2_-), 2.80 (1H, dd, *J*_3eq,3ax_ = 16.8, *J*_3eq,2_ = 2.9 Hz, H-3_eq_), 3.07 (1H, dd, *J*_3ax,3eq_ = 16.7, *J*_3ax,2_ = 13.2 Hz, H-3_ax_), 5.58 (1H, dd, *J*_2,3ax_ = 13.0, *J*_2,3eq_ = 2.8 Hz, H-2), 6.80 (1H, dd, *J*_7,8_ = 8.8 Hz, *J*_7,5_ = 3.0 Hz, H-7), 6.85 (1H, d, *J*_5,7_ = 2.9 Hz, H-5), 7.37 (3H, m, H-3', H-4', H-5'), 7.52 (2H, m, H-2', H-6'), and 7.87 (1H, d, *J*_8,7_ = 8.9 Hz, H-8); ^13^C-NMR (THF-d_8_) δ: 9.3 (CH_3_CH_2_-), 28.2 (CH_3_CH_2_-), 45.2 (C-3), 81.2 (C-2), 111.9 (C-5), 116.4 (C-8), 119.9 (C-10), 127.3 (C-7), 128.8 (C-2', C-6'), 129.4 (C-4'), 129.5 (C-3', C-5'), 140.6 (C-1'), 157.9 (C-6), 163.5 (C-9), 172.3 (>H=0), and 190.5 (C-4).

### 3.2. Biological Methods

#### 3.2.1. Reagents

The compounds (6-HF and 6-PF) were dissolved in DMSO (50 mM) to a final concentration of 0.1% (*v/v*) in the culture media. Soluble recombinant human TRAIL (rhsTRAIL) was purchased from PeproTech Inc. (Rocky Hill, NJ, USA).

#### 3.2.2. Cancer Cells

The experiments were performed on a HeLa human cervical cancer cell line obtained from DSMZ (Deutsche Sammlung von Mikroorganismen und Zellkulturen) GmbH-German Collection of Microorganism and Cell Cultures (Braunschweig, Germany). The HeLa cells were grown in monolayer cultures in RPMI 1640 containing 10% fetal bovine serum with 4 mM L-glutamine, 100 U/mL penicillin and 100 μg/mL streptomycin. The cells were maintained at 37 °C in atmosphere with 5% CO_2_ [25,30]. All reagents for cell culture were purchased from PAA Laboratories (Pasching, Austria).

#### 3.2.3. Detection of Cell Death Using MTT Colorimetric Assay

The cytotoxicity was determined by the 3-(4,5-dimethylthiazol-2-yl)-2,5-diphenyltetrazolium bromide (MTT) assay as described previously [53,54]. The HeLa cells (2.5 × 10^5^/mL) were seeded 24 h before the experiments in a 96-well plate. TRAIL (100 ng/mL) and/or flavanones (50–100 μM) were added to the cells, and 48 h later the medium was removed and 20 μL MTT solutions prepared at 5 mg/mL (Sigma Chemical Company) were added to each well for 4 h. The resulting crystals were dissolved in DMSO. Controls included native cells and medium alone. The spectrophotometric absorbance of each well was measured using a microplate reader (ELx 800, Bio-Tek Instruments Inc., Winooski, VT, USA) at 550 nm. The percent cytotoxicity was calculated by the formula: percent cytotoxicity (cell death) = [1 − (absorbance of experimental wells/absorbance of control wells)] × 100%.

#### 3.2.4. Lactate Dehydrogenase Release Assay

Lactate dehydrogenase (LDH) is a stable cytosolic enzyme released upon membrane damage in necrotic cells. Measurement of LDH activity was performed using a commercial cytotoxicity assay kit (Roche Diagnostics GmbH, Mannheim, Germany), in which LDH is detected in culture supernatants with a coupled enzymatic assay, resulting in conversion of a tetrazolium salt into red formazan product. The HeLa cells were treated with various concentrations of flavanones (50–100 μM) alone and in combination with TRAIL (100 ng/mL) for the indicated period of time. The sample solution (supernatant) was removed and LDH released from cells was measured in culture medium. The maximal release was obtained after treating control cells with 1% Triton X-100 for 10 min at room temperature [55,56]. The necrotic percentage was expressed using the formula: (sample value/maximal release) × 100%.

#### 3.2.5. Determination of Apoptosis by Flow Cytometry with Annexin V-FITC Staining

Apoptosis was determined by flow cytometry using the Apoptest-FITC Kit with annexin V (Dako, Glostrup, Denmark). HeLa cells (2.5 × 10^5^/mL) were seeded in 24-well plates for 24 h prior to experimentation and then exposed to TRAIL (100 ng/mL) and/or flavanones (50–100 μM) for 48 h. After the incubation, the cells were washed twice with phosphate-buffered saline solution (PBS) and resuspended in 500 μL of binding buffer. The cell suspension (290 μL) was then incubated with 5 μL of annexin V-FITC and 5 μL of propidium iodide for 10 min at room temperature in the dark. The population of annexin V-positive cells was evaluated by flow cytometry (BD LSR II Analyzer, Becton Dickinson Immunocytometry Systems, San Jose, CA, USA) [34,57].

#### 3.2.6. Determination of Apoptosis by Fluorescence Microscopy with Annexin V-FITC Staining

Apoptotic cells were quantified by the fluorescence microscopy method using the Apoptotic & Necrotic & Healthy Cells Quantification Kit from Biotium, Inc. (Hayward, CA, USA) [25,30]. The HeLa cells (2.5 × 10^5^/mL) were seeded 24 h before the experiments in a 24-well plate. TRAIL (100 ng/mL) and/or flavanones (50–100 μM) were added to cancer cells, and 48 h later the cells were washed with PBS and detached from cell culture wells by trypsin. Next, the cells were centrifuged to discard supernatant, washed with PBS and resuspended in binding buffer (100 μL/sample). To each tube there were added: 5 μL Annexin V-FITC, 5 μL Ethidium Homodimer III and 5 μL Hoechst 33342 solutions. The samples were incubated at room temperature for 15 min in the dark. After staining the cancer cells were washed with binding buffer and placed on a glass slide and covered with a glass coverslip. The stained cells were observed under a fluorescence inverted microscope IX51 (Olympus, Tokyo, Japan) using filter set for FITC, TRITC and DAPI. The healthy cells (stained with Hoechst 33342) emitted blue fluorescence, apoptotic cells (stained with Annexin V-FITC and Hoechst 33342) emitted green and blue fluorescence and necrotic cells (stained with Ethidium Homodimer III and Hoechst 33342) emitted red and blue fluorescence.

#### 3.2.7. Flow Cytometric Analysis of Death Receptor Expression on the Cancer Cell Surface

The cell surface expression of death receptors TRAIL-R1 and TRAIL-R2 was detected by flow cytometry (LSR II, Becton Dickinson Immunocytometry Systems). HeLa cells (2.5 × 10^5^/mL) were seeded in 24-well plates for 24 h and exposed to flavanones (50 μM) for 48 h. Cells were then harvested using solution of trypsin and ethylenediaminetetraacetic acid, washed twice in PBS and resuspended in PBS containing 0.5% bovine serum albumin. Cells were incubated with 10 μL phycoerythrin-conjugated anti-TRAIL-R1 or anti-TRAIL-R2 monoclonal antibody (R&D Systems, Minneapolis, MN, USA) at 4 °C for 45 min. After staining, the cells were washed with PBS and analysed using flow cytometry [48,58]. The control sample (isotype control) consisted of cells in a separate tube treated with phycoerythrin-labelled mouse IgG_1_ or mouse IgG_2B_ (R&D Systems).

#### 3.2.8. Evaluation of Mitochondrial Membrane Potential by DePsipher

The DePsipher Kit (R&D Systems) was used to measure the mitochondrial membrane potential by fluorescence microscopy [32,35]. HeLa cells (2.5 × 10^5^/mL) were seeded in a 24-well plate 24 h prior to the experiments. TRAIL (100 ng/mL) and/or flavanones (50 μM) were added, and 48 h later, the cells were washed with PBS and harvested by trypsinisation. The cells were incubated in the dark with DePsipher (5,5',6,6'-tetrachloro-1,1',3,3'-tetraethylbenzimidazolyl carbocyanin iodide) solution at a concentration of 5 μg/mL for 30 min at 37 °C, washed with reaction buffer with stabiliser, placed on a glass slide and covered with a glass cover slip. The stained cells were observed with a fluorescence inverted microscope using filter sets for FITC and TRITC. DePsipher undergoes potential-dependent accumulation in the mitochondria, which is indicated by a fluorescence emission shift from red (590 nm) to green (530 nm).

### 3.3. Statistical Analysis

The results are expressed as means ± S.D. obtained from three separate experiments performed in quadruplicate (n = 12). Statistical significance was evaluated using t Student’s test. *p* < 0.05 were considered significant.

## 4. Conclusions

Both synthetic flavanones, 6-HF and its derivative 6-PF, augment TRAIL-induced apoptosis in HeLa cells and sensitize TRAIL-resistant cancer cells by engaging extrinsic and intrinsic apoptotic pathway via up-regulation of TRAIL-R2 expression and induction of ΔΨm loss. The study demonstrates the potential use of 6-HF and 6-PF in TRAIL-based anticancer therapy and prevention.

## References

[1] Androutsopoulos V.P., Papakyriakou A., Vourloumis D., Tsatsakis A.M., Spandidos D.A. (2010). Dietary flavonoids in cancer therapy and prevention: Substrates and inhibitors of cytochrome P450 CYP1 enzymes. Pharmacol. Ther..

[2] Krol W., Shani J., Czuba Z., Scheller S. (1992). Modulation luminol-dependent chemiluminescence of neutrophils by flavones. Z. Naturforsch. C.

[3] Vanamala J., Leonardi T., Patil B.S., Taddeo S.S., Murphy M.E., Pike L.M., Chapkin RS., Lupton J.R., Turner N.D. (2006). Suppression of colon carcinogenesis by bioactive compounds in grapefruit. Carcinogenesis.

[4] Turner N.D., Paulhill K.J., Warren C.A., Davidson L.A., Chapkin R.S., Lupton J.R., Carroll R.J., Wang N. (2009). Quercetin suppresses early colon carcinogenesis partly through inhibition of inflammatory mediators. Acta Hortic..

[5] Warren C.A., Paulhill K.J., Davidson L.A., Lupton J.R., Taddeo S.S., Hong M.Y., Carroll R.J., Chapkin R.S., Turner N.D. (2009). Quercetin may suppress rat aberrant crypt foci formation by suppressing inflammatory mediators that influence proliferation and apoptosis. J. Nutr..

[6] Patil B.S., Jayaprakasha G.K., Chidambara-Murthy K.N., Vikram A. (2009). Bioactive compounds: Historical perspectives, opportunities and challenges. J. Agric. Food Chem..

[7] Leonardi T., Vanamala J., Taddeo S.S., Davidson L.A., Murphy M.E., Patil B.S., Wang N., Carroll R.J., Chapkin R.S., Lupton J.R. (2010). Apigenin and naringenin suppress colon carcinogenesis through the aberrant crypt stage in azoxymethane-treated rats. Exp. Biol. Med..

[8] Szliszka E., Skaba D., Czuba Z.P., Krol W. (2011). Inhibition of inflammatory mediators by neobavaisoflavone in activated RAW264. 7 macrophages. Molecules.

[9] Barros L., Duenas M., Carvalho A.M., Ferreira I.C., Santos-Buelga C. (2012). Characterization of phenolic compounds in flowers of wild medicinal plants from Northeastern Portugal. Food Chem. Toxicol..

[10] Pinela J., Barros L., Carvalho A.M., Ferreira I.C. (2012). Nutritional composition and antioxidant activity of four tomato (*Lycopersicon esculentum* L.) farmer’ varieties in Northeastern Portugal home gardens. Food Chem. Toxicol..

[11] Lee J.Y., Huerta-Yepez S., Vega M., Baritaki S., Spandidos D.A., Bonavida B. (2007). The NO TRAIL to YES TRAIL in cancer therapy. Int. J. Oncol..

[12] Holoch P.A., Griffith T.S. (2009). TNF-related apoptosis-inducing ligand (TRAIL): A new path to anti-cancer therapies. Eur. J.Pharmacol..

[13] Szliszka E., Mazur B., Zydowicz G., Czuba Z.P., Krol W. (2009). TRAIL-induced apoptosis and expression of death receptor TRAIL-R1 and TRAIL-R2 in bladder cancer cells. Folia Histochem. Cytobiol..

[14] Mellier G., Huang S., Shenoy K., Pervaiz S. (2010). TRAILing death in cancer. Mol. Aspects Med..

[15] Russo M., Mupo A., Spagnuolo C., Russo G.L. (2010). Exploring death receptor pathways as selective targets in cancer therapy. Biochem. Pharmacol..

[16] Szliszka E., Krol W. (2011). The role of dietary polyphenols in tumor necrosis factor-related apoptosis inducing ligand (TRAIL)-induced apoptosis for cancer chemoprevention. Eur. J. Cancer Prev..

[17] Szliszka E., Czuba Z.P., Bronikowska J., Mertas A., Paradysz A., Krol W. (2011). Ethanolic extract of propolis (EEP) augments TRAIL-induced apoptotic death in prostate cancer cells. Evid. Based Complement. Alternat. Med..

[18] Szliszka E., Zydowicz G., Janoszka B., Dobosz C., Kowalczyk-Ziomek G., Krol W. (2011). Ethanolic extract of Brazilian green propolis sensitizes prostate cancer cells to TRAIL-induced apoptosis. Int. J. Oncol..

[19] Zhang L., Fang B. (2005). Mechanisms of resistance to TRAIL-induced apoptosis in cancer. Cancer Gene Ther..

[20] Horinanka M., Yoshida T., Shiraishi T., Nakata S., Wakada M., Nakanishi R., Nishino H., Sakai T. (2005). The combination of TRAIL and luteolin enhances apoptosis in human cervival cancer HeLa cells. Biochem. Biophys. Res. Comun..

[21] Horinaka M., Yoshida T., Shiraishi T., Nakata S., Wakada M., Sakai T. (2006). The dietary flavonoid apigenin sensitizes malignant tumor cells to tumor necrosis factor-related apoptosis-inducing ligand. Mol. Cancer Ther..

[22] Son Y.G., Kim E.H., Kim J.Y., Kim S.U., Kwon T.K., Yoon A.R., Yun C.O., Choi K.S. (2007). Silibinin sensitizes human glioma cells to TRAIL-mediated apoptosis via DR5 upregulation and downregulation of cFLIP and surviving. Cancer Res..

[23] Chen W., Wang X., Zhuang J., Zhang L., Lin Y. (2007). Induction of death receptor 5 and suppression of surviving contribute to sensitization of TRAIL-induced cytotoxicity by quercetin in non-small lung cancer cells. Carcinogenesis.

[24] Kim J.Y., Kim E.H., Park S.S., Lim J.H., Kwon T.K., Choi K.S. (2008). Quercetin sensitizes human hepatoma cells to TRAIL-induced apoptosis via Sp1-mediated DR5 upregulation and proteasome-mediated c-FLIPS downregulation. J. Cell. Biochem..

[25] Szliszka E., Czuba Z.P., Jernas K., Krol W. (2008). Dietary flavonoids sensitize HeLa cells to tumor necrosis factor-related apoptosis-inducing ligand (TRAIL). Int. J. Mol. Sci..

[26] Yoshida T., Konishi M., Horinaka M., Yasuda T., Goda A.E., Taniguchi H., Yano K., Wakada M., Sakai T. (2008). Kaempferol sensitizes colon cancer cells to TRAIL-induced apoptosis. Biochem. Biophys. Res. Commun..

[27] Tanaguchi H., Yoshida T., Horinaka M., Yasuda T., Goda A.E., Konishi M., Wakada M., Kataoka K., Yoshikawa T., Sakai T. (2008). Baicalein overcomes tumor necrosis factor-related apoptosis-inducing ligand resistance via two different cell specific pathways in cancer cells but not in normal cells. Cancer. Res..

[28] Jung Y.H., Heo J., Lee Y.J., Kwon T.K., Kim Y.H. (2010). Quercetin enhances TRAIL-induced apoptosis in prostate cancer cells via increased protein stability of death receptor 5. Life Sci..

[29] Szliszka E., Gebka J., Bronikowska J., Krol W. (2010). Dietary flavones enhance the effect of tumor necrosis factor-related apoptosis-inducing ligand (TRAIL) on bladder cancer cells. Cent. Eur. J. Urol..

[30] Bronikowska J., Szliszka E., Czuba Z.P., Zwolinski D., Szmydki B., Krol W. (2010). The combination of TRAIL and isoflavones enhances apoptosis in cancer cells. Molecules.

[31] Szliszka E., Czuba Z.P., Mazur B., Sedek L., Paradysz A., Krol W. (2010). Chalcones enhance TRAIL-induced apoptosis in prostate cancer cells. Int. J. Mol. Sci..

[32] Szliszka E., Czuba Z.P., Mazur B., Paradysz A., Krol W. (2010). Chalcones and dihydrochalcones augment TRAIL-mediated apoptosis in prostate cancer cells. Molecules.

[33] Szliszka E., Bronikowska J., Czuba Z.P., Krol W. (2010). Isoflavones augment the effect of tumor necrosis factor-related apoptosis-inducing ligand (TRAIL) on prostate cancer cells. Cent. Eur. J. Urol..

[34] Szliszka E., Helewski K.J., Mizgala E., Krol W. (2011). The dietary flavonol fisetin enhances the apoptosis-inducing potential of TRAIL in prostate cancer cells. Int. J. Oncol..

[35] Szliszka E., Krol W. (2011). Soy isoflavones augment the effect of TRAIL-mediated apoptotic death in prostate cancer cells. Oncol. Rep..

[36] Jin C.Y., Park C., Hwang H.J., Kim G.Y., Choi B.T., Kim W.J., Choi Y.H. (2011). Naringenin up-regulates the expression of death receptor 5 and enhances TRAIL-induced apoptosis in human lung cancer A549 cells. Mol. Nutr. Food Res..

[37] Szliszka E., Czuba Z.P., Mertas A., Paradysz A., Krol W. (2011). The dietary isoflavone biochanin-A sensitizes prostate cancer cells to TRAIL-induced apoptosis. Urol. Oncol..

[38] Wudtiwai B., Sripanidkulchai B., Kongtawelert P., Banjerdpongchai R. (2011). Metoxyflavone derivatives modulates the effect of TRAIL-induced apoptosis in human leukemic cell lines. J. Hematol. Oncol..

[39] Ding J., Polier G., Köhler R., Giaisi M., Krammer P.H., Li-Weber M. (2012). Wogonin and related natural flavones overcome tumor necrosis factor-related apoptosis inducing ligand (TRAIL) protein resistance of tumors by down-regulation of c-FLIP protein and up-regulation of TRAIL receptor 2 expression. J. Biol. Chem..

[40] Graf B.A., Milbury P.E., Blumberg J.B. (2005). Flavonols, flavones and flavanones, and human health: Epidemiological evidence. J. Med. Food.

[41] Park J.H., Jin C.Y., Lee B.K., Kim G.Y., Choi Y.H., Jeong Y.K. (2008). Naringenin induces apoptosis through downregulation of Akt and caspase-3 activation in human leukemia THP-1 cells. Food Chem. Toxicol..

[42] Jin C.Y., Park C., Lee J.H., Chung K.T., Kwon T.K., Kim G.Y., Choi B.T., Choi Y.H. (2009). Naringenin-induced apoptosis is attenuated by Bcl-2 but restored by small molecule Bcl-2 inhibitor, HA 14–1 in human leukemia U977 cells. Toxicol. In Vitro.

[43] Park H.J., Kim M.J., Ha E., Chung J.H. (2008). Apoptotic effect of hesperidin through caspase-3 activation in human colon cells SNU-C4. Phytomedicine.

[44] Ghorbani A., Nazari M., Jeddi-Tehrani M., Zang H. (2012). The citrus flavonoid hesperidin induces p53 and inhibits NF-κB activation in order to trigger apoptosis in NALM-6 cells: Involvement of PPARγ-dependent mechanism. Eur. J. Nutr..

[45] Zhang S., Zhou Y., Liu Y., Cai Y. (2009). Effect of liquiritigenin, a flavone existed from *Radix*
*glycyrrhizae* on pro-apoptotic in SMMC-7721 cells. Food Chem. Toxicol..

[46] Liu C., Wang Y., Xie S., Zhou Y., Ren X., Li X., Cai Y. (2011). Liquiritigenin induces mitochondria-mediated apoptosis via cytochrome c release and caspases activation in HeLa cells. Phytother. Res..

[47] Ko C.H., Shen S., Chen Y. (2004). Hydroxylation at C4' or C6 is essential for apoptosis-inducing activity of flavanone through activation of the casapase-3 cascade and production of reactive oxygen species. Free Radic. Biol. Med..

[48] Szliszka Ewelina. Medical University of Silesia, Katowice, ON. Unpublished work, 2012.

[49] Bronikowska J., Szliszka E., Jaworska D., Czuba Z.P., Krol W. (2012). The coumarin psoralidin enhances anticancer effect of tumor necrosis factor-related apoptosis-inducing ligand (TRAIL). Molecules.

[50] Jacquemin G., Granci V., Gallouet A.S., Lalaoui N., Morle A., Iessi E., Morizot A., Garrido C., Guillaudeux T., Micheau O. (2012). Quercetin-mediated Mcl-1 and surviving downregulation restores TRAIL-induced apoptosis in non-Hodgkin’s lymphoma B cells. Haematologica.

[51] Kostrzewa-Susłow E., Dmochowska-Gładysz J., Białońska A., Ciunik Z., Rymowicz W. (2006). Microbial transformations of flavanone and 6-hydroxyflavanone by *Aspergillus niger* strains. J. Mol. Catal. B Enzym..

[52] Kostrzewa-Susłow E., Białońska A., Janeczko T. (2010). 4-Oxo-2-phenylchroman-6-yl propionate. Acta Cryst..

[53] Szliszka E., Majcher A., Domino M., Pietsz G., Krol W. (2007). Cytotoxic activity of tumor necrosis factor-related apoptosis-inducing ligand (TRAIL) against bladder cancer cells after using chemotherapeutic drugs. Urol. Pol..

[54] Szliszka E., Bronikowska J., Majcher A., Miszkiewicz J., Krol W. (2009). Enhanced sensitivity of hormone-refractory prostate cancer cells to tumor necrosis factor-related apoptosis-inducing ligand (TRAIL) mediated cytotoxicity by taxanes. Cent. Eur. J. Urol..

[55] Szliszka E., Czuba ZP., Domino M., Mazur B., Zydowicz G., Krol W. (2009). Ethanolic extract of propolis (EEP) enhances the apoptosis-inducing potential of TRAIL in cancer cells. Molecules.

[56] Szliszka E., Czuba Z.P., Sedek L., Paradysz A., Krol W. (2011). Enhanced TRAIL-mediated apoptosis in prostate cancer cells by the bioactive compounds neobavaisoflavone and psoralidin isolated from *Psoralea corylifolia*. Pharmacol. Rep..

[57] Szliszka E., Czuba Z.P., Kawczyk-Krupka A., Sieron-Stoltny A., Sieron A., Krol W. (2012). Chlorin-based photodynamic therapy enhances the effect of tumor necrosis factor-related apoptosis-inducing ligand (TRAIL) in bladder cancer cells. Med. Sci. Monit..

[58] Szliszka E., Zydowicz G., Mizgala E., Krol W. (2012). Artepillin C (3,5-diprenyl-4-hydroxycinnamic acid) sensitizes prostate cancer LNCaP cells to TRAIL-induced apoptosis. Int. J. Oncol..

